# Automated Process for Monitoring of Amiodarone Treatment: Development and Evaluation

**DOI:** 10.2196/65473

**Published:** 2025-02-19

**Authors:** Birgitta I Johansson, Jonas Landahl, Karin Tammelin, Erik Aerts, Christina E Lundberg, Martin Adiels, Martin Lindgren, Annika Rosengren, Nikolaos Papachrysos, Helena Filipsson Nyström, Helen Sjöland

**Affiliations:** 1 Department of Molecular and Clinical Medicine Sahlgrenska Academy University of Gothenburg Gothenburg Sweden; 2 Department of Medicine, Geriatrics and Emergency Medicine Sahlgrenska University Hospital Gothenburg Sweden; 3 Department of Digital Development Sahlgrenska University Hospital Gothenburg Sweden; 4 Department of Endocrinology Sahlgrenska University Hospital Gothenburg Sweden; 5 Department of Internal Medicine and Clinical Nutrition Institute of Medicine University of Gothenburg Gothenburg Sweden; 6 Wallenberg Centre for Molecular and Translational Medicine Gothenburg Sweden; 7 Chalmers University of Technology Gothenburg Sweden; 8 Department of Food and Nutrition, and Sport Science Faculty of Education University of Gothenburg Gothenburg Sweden; 9 School of Public Health and Community Medicine Institute of Medicine University of Gothenburg Gothenburg Sweden; 10 Gothenburg’s Centre for Person-Centred Care University of Gothenburg Gothenburg Sweden

**Keywords:** thyroid function, robotics, follow-up studies, disease management, decision support, automated process, monitoring, amiodarone treatment, anti-arrhythmic medication, anti-arrhythmic, development, evaluation, thyroid, liver, side effects, cardiac dysrhythmias, ventricular tachycardia, ventricular fibrillation, arrhythmia, automation, robot, algorithm, clinical decision support system, thyroid gland, heart, atrial fibrillation

## Abstract

**Background:**

Amiodarone treatment requires repeated laboratory evaluations of thyroid and liver function due to potential side effects. Robotic process automation uses software robots to automate repetitive and routine tasks, and their use may be extended to clinical settings.

**Objective:**

Thus, this study aimed to develop a robot using a diagnostic classification algorithm to automate repetitive laboratory evaluations for amiodarone follow-up.

**Methods:**

We designed a robot and clinical decision support system based on expert clinical advice and current best practices in thyroid and liver disease management. The robot provided recommendations on the time interval to follow-up laboratory testing and management suggestions, while the final decision rested with a physician, acting as a human-in-the-loop. The performance of the robot was compared to the existing real-world manual follow-up routine for amiodarone treatment.

**Results:**

Following iterative technical improvements, a robot prototype was validated against physician orders (n=390 paired orders). The robot recommended a mean follow-up time interval of 4.5 (SD 2.4) months compared to the 3.1 (SD 1.4) months ordered by physicians (*P<*.001). For normal laboratory values, the robot recommended a 6-month follow-up in 281 (72.1%) of cases, whereas physicians did so in only 38 (9.7%) of cases, favoring a 3- to 4-month follow-up (n=227, 58.2%). All patients diagnosed with new side effects (n=12) were correctly detected by the robot, whereas only 8 were by the physician.

**Conclusions:**

An automated process, using a software robot and a diagnostic classification algorithm, is a technically and medically reliable alternative for amiodarone follow-up. It may reduce manual labor, decrease the frequency of laboratory testing, and improve the detection of side effects, thereby reducing costs and enhancing patient value.

## Introduction

Amiodarone is a potent antiarrhythmic drug widely used for both severe supraventricular and ventricular arrhythmias. However, careful follow-up is necessary during treatment due to potentially serious adverse effects and toxic reactions in the thyroid gland, liver, lung, and heart. Approximately 15% of patients will experience adverse effects during the first year of amiodarone use and 50% during long-term treatment, most often thyroid and liver dysfunction [1]. Reported frequencies of amiodarone-induced hypothyroidism (AIH) and amiodarone-induced thyrotoxicosis (AIT) vary between 1%-32% and 0.8%-37.8%, respectively [2-4], with hypothyroidism typically occurring sooner than hyperthyroidism. A careful study showed median time-to-onset at 183 days for AIH and 720 days for AIT [2]. In addition, AIT is often more resistant to treatment due to the complex effects of amiodarone on the thyroid involving hyperthyroidism, thyroiditis, and iodine overload. Hepatotoxicity, with slight elevations of serum transaminase levels, occurs in about 25% of patients whereas hepatitis, cirrhosis, or hepatic failure occurs in less than 3% [5,6].

Amiodarone carries electrophysiological characteristics of all Vaughan Williams classes, although most importantly class 3, which consists of prolongation of the third phase of the action potential in myocardial cells [7]. It is an iodinated benzofuran derivative that is structurally similar to thyroid hormones. It contains about 37% organic iodine, resulting in 40 to 100-fold iodine excess into the systemic circulation [4,8], overloading the thyroid. Amiodarone accumulates in adipose tissue, due to its high lipid solubility, and in highly perfused organs such as the liver, lung, and skin [6]. These properties of amiodarone contribute to the considerable excess risk of primarily thyroid and liver side effects. Additionally, due to the prolonged biological half-life of amiodarone, up to 40-142 days, toxicity can occur in the thyroid and lung even after the treatment is stopped, thus requiring follow-up after discontinuation of therapy [3,8].

Monitoring recommendations during amiodarone treatment according to the North American Society of Pacing and Electrophysiology include thyroid and liver function tests at baseline and every 6 months, supplemented with recurrent radiographic and functional testing [9]. The American Thyroid Association recommends analysis of thyroid-stimulating hormone (TSH) and thyroxine levels at baseline and thereafter every 3-6 months [10]. The most recent updated consensus management guide recommendations, by Epstein et al [1], are in accordance with the North American Society of Pacing and Electrophysiology, proposing follow-up every 3-6 months for the first year and every 6 months thereafter.

Repeated notifications to patients on amiodarone for necessary follow-ups with laboratory testing, examinations, evaluations, and prescriptions for continued medication is a time-consuming and repetitive process. To ensure that no patients are missed, clinical controls with patient-doctor meetings and systematic laboratory follow-ups of side effects are organized independently. Thus, if patients fail to appear or postpone doctor’s meetings they will still be on the administrative waiting list for amiodarone laboratory controls and be regularly summoned for testing. The overall administrative workload for health care professionals is considerable, contributing to workplace stress and a shortage of available personnel. The development of streamlined automated work processes is therefore an attractive option for amiodarone treatment follow-up.

In this study, we present a model for the automation of repetitive follow-up of patients with amiodarone treatment. First, we constructed an automated solution including the whole surveillance process, using a robotic process automation (RPA) software tool. We then compared the RPA to standard manual amiodarone follow-up as presently practiced.

## Methods

### Patients

All patients on, or who recently finished amiodarone treatment (N=198), followed at the outpatient clinic of the Department of Cardiology, Sahlgrenska University Hospital/Östra for surveillance, were included in this study. Clinical background data were collected from patient records and stratified by the type of arrhythmia constituting indication for therapy. All patients received annual in-office appointments with a cardiologist for their clinical condition. Complementary follow-up with laboratory testing owing to the amiodarone treatment was carried out according to routine, irrespective of clinical follow-up. Before the start of the therapy, the standard clinical routine at Sahlgrenska University Hospital/Östra was performed, including the following: thyroid function tests with TSH, free thyroxine, free tri-iodothyronine, liver function tests for aspartate aminotransferase, alanine aminotransferase, ECG, chest x-ray, and a pulmonary function test, including diffusion capacity. After initiation of amiodarone treatment, updated thyroid and liver laboratory blood tests were performed at 3 months, 6 months, and thereafter routinely every 6 months as per local guidelines [1,10], or at the discretion of the cardiologist. The routine further prescribed a complementary test of TSH receptor antibodies or thyroid peroxidase antibodies when a thyroid function test was abnormal. Patients who developed AIT were referred to an endocrinologist, whereas patients who presented with AIH were referred to primary care. Side effects already present at baseline as well as those sustained during follow-up were recorded.

### Process Mapping in the Design of the Robot

**Figure 1:**
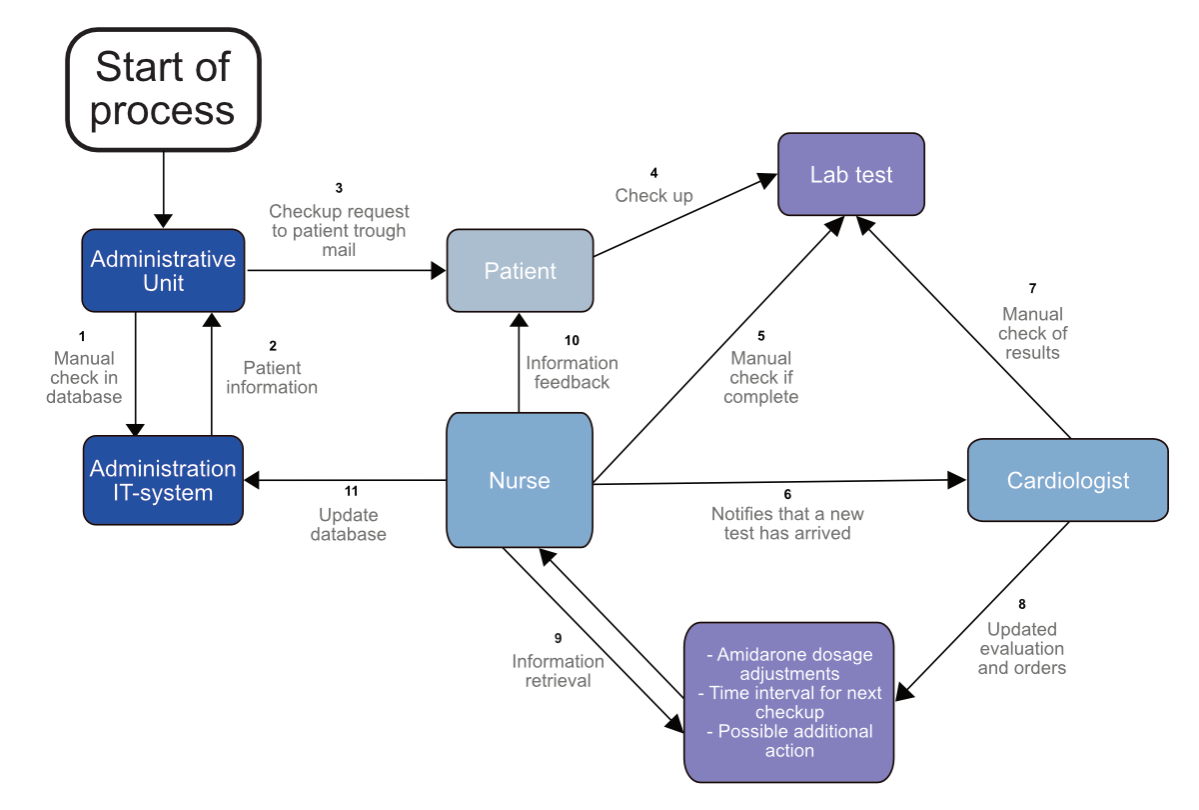
Process map of established manual workflow and steps of the laboratory amiodarone surveillance process. lab: laboratory.

To define the actions that should be included in the RPA procedure, all steps of the laboratory thyroid and liver function surveillance process were mapped by a multiprofessional team (specialized cardiology nurse, cardiologist, administrative personnel, and solution architect; March 2021) and presented in Figure 1. In brief, the manual existing routine in use included the following: (1) patients were manually identified by secretarial personnel from an administrative listing (“amiodarone controls”); (2) orders for blood tests were issued and a time slot for sampling was sent by post to the patients; (3) arrival of completed laboratory analyses were manually monitored from patient records by nurses, and notifications sent to a cardiology specialist; (4) after reviewing the laboratory results the cardiologist decided and recorded the future dose of amiodarone, time interval to next follow-up and possible actions depending on side effects; and (5) the orders were manually transferred to a nurse for execution and notification of the patient. For absent laboratory results the nurse issued a reminder to the patient. This process was typically repeated 2-4 times a year per patient. This routine was maintained during the whole study and included all physicians’ orders assembled in the following analysis, that is, the manual routine constituted the real-world follow-up, and was compared to the robot.

### Automation Tool

A commercially available RPA was programmed to perform tasks following a predetermined set of rules and interact with various information systems (UiPath), thereby performing structured and repetitive tasks quickly and reducing the need for manual human work [11]. It thus mimicked human behavior in repetitive and rule-based work [12], and operated by mapping a process in the RPA tool language for the software robot to follow, with runtime allocated to execute the script by a control dashboard [13].

### Construction of Initial Prototype

Based on the process map, an initial prototype was constructed. The patient administrative system (ELVIS, Insieme Consulting AB) with the acquisition of listed patients for “amiodarone controls,” and the laboratory system (LabBest, CareDialog AB) for access to laboratory results, were integrated. Additionally, an algorithm with reference values for TSH, free thyroxine, free tri-iodothyronine, aspartate aminotransferase, and alanine aminotransferase was built into the process. Three color-coded classes, corresponding to the level of urgency, were created based on reference values. The output was designed as a recommendation for a decision-making cardiologist, serving as a human-in-the-loop, indicating one of three possible actions for each output: class 1: overall normal outcome values (green color) recommending next follow-up test in 6 months, class 2: minor deviation from normal values of uncertain significance (yellow color), recommending follow-up testing in 1 month, and class 3: definite laboratory pathology indicating clear side effect (red color), recommending prompt management by a physician, along with detailed recommendations for management (dose adjustment, discontinuation of amiodarone medication, or consultation of a specific specialist).

### Iterative Improvement of Initial Prototype

The first RPA algorithm (robot 1) was tested by comparing the automated output with the real-world manual procedure from April 2021 to December 2021, including iterative adjustments for clinical optimization. Importantly, the new algorithm incorporated expert clinical advice developed by experienced specialists, 2 endocrinologists (HFN and KT) and 1 gastroenterologist (NP), ensuring state-of-the-art recommendations for assessing and managing both normal and pathological findings. Simultaneous technical adjustments optimized performance and reliability. The resulting algorithm was used for validation studies in the final prototype (robot 2) and is presented and explained in detail in Multimedia Appendix 1.

### Final Prototype for Validation

For validation and comparative testing, a final prototype, robot 2, was constructed based on the aforementioned adjustments. For comparative analyses, we defined robot 2 as “ground truth,” that is, constituting the accurate data used as a reference to validate the system, comparing endocrinologist and gastroenterologist experts’ clinical advice against real-world handling by a cardiologist. We considered experienced endocrinologists and gastroenterologists to have more expertise in hormonal or hepatological evaluations compared to the general cardiologist. Furthermore, the output from robot 2 included written instructions for abnormal findings and is presented as supplementary information including specific clinical routines and references to detailed guidelines (Multimedia Appendix 1). The final prototype, robot 2, was tested from February 2022 to February 2023. Notably, the existing routine process remained in operation during the whole period, with the manual routine executed without awareness of the robot, such that the physician’s orders constituted the real-world outcome and robot 2 worked in parallel producing outcomes only available to the research team. Paired orders, from the physician and robot 2, were compared.

### Comparative Analysis of the Final Prototype for Validation

The comparative analyses’ primary outcomes were the following: (1) the time interval between recommended laboratory testing and (2) the detection of side effects. The distribution of recommended time intervals for follow-up and disagreements between robot and physician were analyzed. First, a strict comparison between the number of months to the next follow-up ordered by the robot and the physician was made. Second, each order made by the physician was classified and labeled per robot 2, as follows: after completion of the test period, all dates for the paired test were retrieved from the robot and laboratory results and patient records were examined by one of the authors (HS) and classified as correct or incorrect according to the rules of the algorithm, when compared to the decision made by the physician responsible for managing the case. This evaluation was made without knowledge of the robot classifications. If patient records were ambiguous consultations were made with another author from the team (BIJ). The results from robot classifications were then retrieved for the pairing of outcome classifications and comparative analysis.

We used a confusion matrix as a performance estimate of the physician’s classification compared to the robot. A confusion matrix is a statistical tool used to evaluate the performance of a classification model. In this context, it was used to visualize the accuracy of the physician-predicted side effects based on the laboratory test results, comparing the physician-derived orders with the orders by robot 2. The labels applied on classification were the following: (1) true positive (TP) for pathological laboratory results (corresponding to class 2-3, yellow or red), (2) true negative (TN) for normal laboratory results (corresponding to class 1, green), (3) false positive (FP) for follow-up tests ordered by the physician for a time interval >1 month shorter than that ordered by robot 2 (ie, judged as more urgent than ground truth by the physician), and (4) false negative (FN), follow-up tests ordered for a time interval >1 month longer than that ordered by robot 2 (ie, judged as less urgent than ground truth by the physician) or when a definitely pathological test result was not noticed by the physician. Paired orders were compared (n=390 observations). All labels given by the robot and physician were combined into a data frame and presented in the confusion matrix. If the time interval to follow-up test between the algorithm and physician differed ≤1 month the same labels were given. As robot 2 was judged as ground truth, the confusion matrix is limited to true labels (TP and TN) for actual outcomes.

### Analysis of Differing Classifications Between the Robot and Physician

To further compare robot 2 and physician estimations, we analyzed the distribution of months to the next follow-up. To investigate which classifications (classes 1-3) contributed the most to the disagreements, that is, the false classifications observed in the confusion matrix, all observations were grouped based on the 3 robot classifications and sorted according to the labels given in the confusion matrix. If the label given by the physician matched the label given by robot 2 the observation was judged as correct. If the physician had given any label other than robot 2, the observation was considered to be incorrect. Classes 1-3 were converted to a month-based scale. Outcome class 3 (red color) was classified as “0 month” since red represented the highest urgency calling for “directly to physician for handling.”

### Detection of Side Effects

For patients who suffered side effects diagnosed in the patient record during this study, we compared the likelihood of detection between robot 2 and the physician. For robot 2, the correct detection of a side effect was judged as class 3 (red). The correct response to a side effect by the physician (comparable to class 3 [red]), was defined as at least one of the following noted in the patient record: (1) the diagnostic side effect was acknowledged by the cardiologist, (2) the indication for amiodarone was reevaluated, (3) adjustment or termination of medication was undertaken, or (4) referral or consultation with the appropriate specialist was undertaken. Incorrect or questionable action (yellow) was assessed when a new laboratory test was carried out without any of the aforementioned actions.

### Statistical Analysis

Data are presented as numbers and percentages, mean (SD) for normally distributed variables tested with the Shapiro-Wilk test or median (IQR), unless otherwise stated. For comparison between groups, the Wilcoxon signed rank test was used. The demographic data includes the population participating during the robot 2 evaluation period.

### Ethical Considerations

This study was performed per the principles of the Declaration of Helsinki. The project was approved by the Swedish Ethical Review Authority (2021-01962). This study was observational and informed consent was waived, as the presented robot 2 outcome represents a simulation, and the regular manual routine was upheld.

## Results

### Demographics of Cohort

Between April 9, 2021, and February 28, 2023, a total of 198 patients, with a mean age of 73 (SD 12) years and 62.6% (n=124) men, were under follow-up routines due to amiodarone prescription and were included in this study. Indications for amiodarone were atrial (n=145, 73.2%) or ventricular (n=53, 26.8%) arrhythmias. The most frequent comorbidities were heart failure (n=142, 71.7%), hypertension (n=127, 64.1%), and coronary artery disease (n=67, 33.8%). Of patients with atrial or ventricular arrhythmias, 23.4% (n=34) and 41.5% (n=22), respectively, had a left ventricular ejection fraction <40%. At baseline, 79.8% (n=156) were on β-blockers, 62.6% (n=124) on novel oral anticoagulants, and 17.2% (n=34) on thyroid hormone (levothyroxine; Table 1).

**Table 1:** Baseline data for patients with amiodarone treatment at the start of this study^a^.

Variables					All patients					Atrial arrhythmia								Ventricular arrhythmia
Population, n (%)					198 (100)					145 (73.2)								53 (26.8)
**Demographics**																		
	Male, n (%)			124 (62.6)					87 (60)					37 (69.8)				
	Age (years), mean (SD)			73 (12)					72 (13)					75 (9)				
	BMI, median (IQR)			27 (24-31)					27 (24-31)					26 (24-31)				
	Smoking, n (%)			11 (5.5)					9 (6.2)					2 (3.7)				
**Comorbidity, n (%)**																		
	Hypertension		127 (64.1)						94 (64.8)				33 (62.3)					
	Diabetes type I		3 (1.5)						1 (0.7)				2 (3.8)					
	Diabetes type II		33 (16.7)						20 (13.8)				13 (24.5)					
	Dyslipidemia		28 (14.1)						25 (17.2)				3 (5.7)					
	Coronary artery disease		67 (33.8)						42 (29)				25 (47.2)					
	Congenital heart disease		9 (4.5)						8 (5.5)				1 (1.9)					
	COPD^b^		11 (5.6)						6 (4.1)				5 (9.4)					
	Chronic kidney disease		24 (12.1)						17 (12)				7 (13.2)					
	Stroke		31 (15.7)						19 (13.1)				12 (22.6)					
	Thyroid disease		17 (8.6)						11 (7.6)				6 (11.3)					
	Heart failure		142 (71.7)						97 (66.9)				45 (84.9)					
	HFpEF^c^		54 (27.2)						44 (30.3)				10 (18.9)					
	HFmrEF^d^		32 (16.2)						19 (13.1)				13 (24.5)					
	HFrEF^e^		56 (28.3)						34 (23.4)				22 (41.5)					
**Medication, n (%)**																		
	β-blockers						156 (79.8)				109 (75.2)					47 (88.7)		
	Digitalis						4 (2)				3 (2.1)					1 (1.9)		
	Calcium channel blockers						45 (22.7)				32 (22.1)					13 (24.5)		
	ACEI-ARB^f^						96 (48.5)				73 (50.3)					23 (43.4)		
	ARNI^g^						41 (20.7)				24 (16.6)					17 (32.1)		
	Mineralocorticoid receptor antagonist						74 (37.4)				48 (33.1)					26 (49.1)		
	Acetylsalicylic acid						24 (12.1)				9 (6.2)					15 (28.3)		
	Platelet inhibitors, other						15 (7.6)				5 (3.4)					10 (18.9)		
	Novel oral anticoagulant						124 (62.6)				107 (73.8)					17 (32.1)		
	Waran						33 (16.7)				24 (16.6)					9 (17)		
	Heparin						3 (1.5)				3 (2.1)					0 (0)		
	Metformin						22 (11.1)				13 (9)					9 (17)		
	SGLT2^h^-inhibitor						25 (12.6)				16 (11)					9 (17)		
	Insulin						9 (4.5)				5 (3.4)					4 (7.5)		
	Statins						105 (53)				73 (50.3)					32 (60.4)		
	Long-acting nitroglycerine						16 (8.1)				8 (5.5)					8 (15.1)		
	Diuretics						75 (37.9)				53 (36.6)					22 (41.5)		
	Thyroid hormone (levothyroxine)						34 (17.2)				23 (15.9)					11 (20.8)		
**X-ray contrast** **(in prior history)**																		
	Exposure, n (%)					173 (87.4)			122 (84.1)						51 (96.2)			
	Exposure number of times, median (IQR)					2 (2-4)			2 (2-4)						3 (2-4)			
**Laboratory values**																		
	Thyroid-stimulating hormone (mIU^i^/L), median (IQR)	1.9 (1.3-2.8)						1.9 (1.2-2.7)				2.1 (1.2-2.7)						
	Free thyroxin (pmol/L), median (IQR)			15 (13-17)					15 (13-17)								14 (12-17)	
	AST^j^ (µkat/L), median (IQR)			0.44 (0.36-0.56)					0.45 (0.39-0.54)								0.44 (0.34-0.61)	
	ALT^k^ (µkat/L), median (IQR)			0.4 (0.29-0.63)					0.41 (0.3-0.61)								0.39 (0.25-0.64)	
**Left ventricular ejection fraction**																		
	LVEF^l^, median (IQR)			50 (40-59)					55 (45-60)					45 (30-55)				
	LVEF <40%, n (%)			44 (22.2)					24 (16.6)					20 (37.7)				

^a^Values are presented as the number of observations (percentage of population) if not otherwise specified.

^b^COPD: chronic obstructive pulmonary disease.

^c^HFpEF: heart failure with preserved ejection fraction.

^d^HFmrEF: heart failure with midrange ejection fraction.

^e^HFrEF: heart failure with reduced ejection fraction.

^f^ACEI-ARB: angiotensin converter enzyme inhibitors or angiotensin receptor blockers.

^g^ARNI: angiotensin receptor neprilysin inhibitor.

^h^SGLT2: sodium-glucose transport protein 2.

^i^IU: international units.

^j^AST: aspartate aminotransferase.

^k^ALT: alanine aminotransferase.

^l^LVEF: left ventricular ejection fraction.

### Construction of the Prototype for Validation

**Figure 2:**
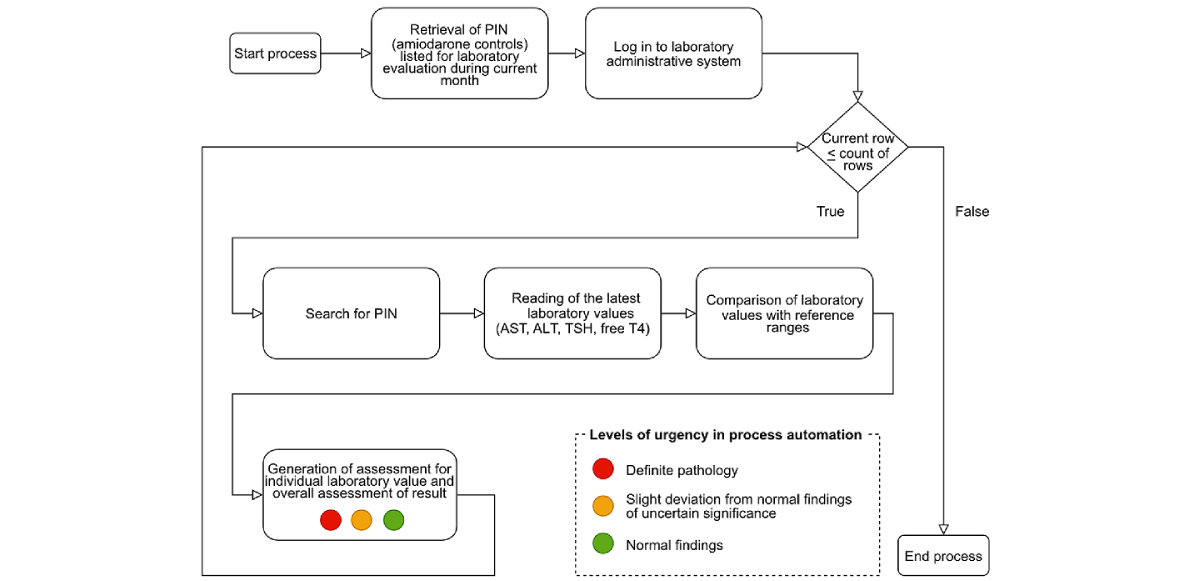
Process map of workflow and procedures included in the prototype robot (robot 2). ALT: alanine aminotransferase; AST: aspartate aminotransferase; PIN: personal identification number; T4: thyroxin; TSH: thyroid stimulating hormone.

**Figure 3:**
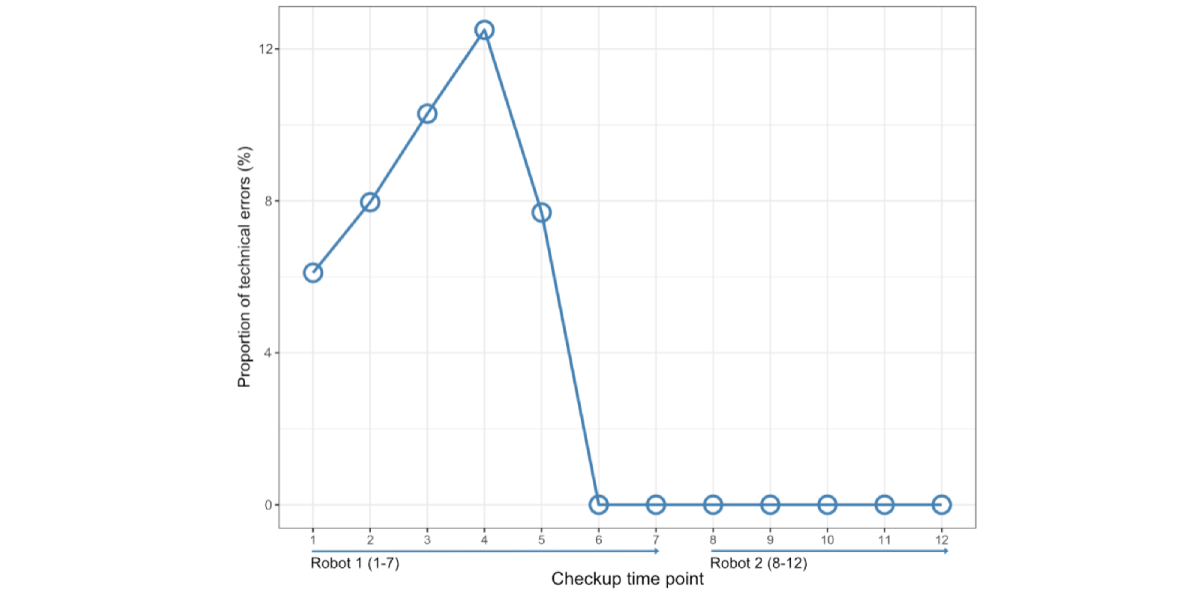
Proportion of technical robot errors over time (checkup time points).

The first prototype, robot 1, was developed from April 2021 to December 2021 through iterative adjustments, eliminating technical errors and improving the medical algorithm until obtaining satisfactory performance as judged by the multiprofessional team, resulting in the final prototype for validation, robot 2 (Figure 2). The technical improvement and reduction of errors over time are presented, with an error defined as an output from the robot not resulting in classification 1, 2, or 3 (Figure 3).

### Comparative Analysis of the Final Prototype for Validation

**Figure 4:**
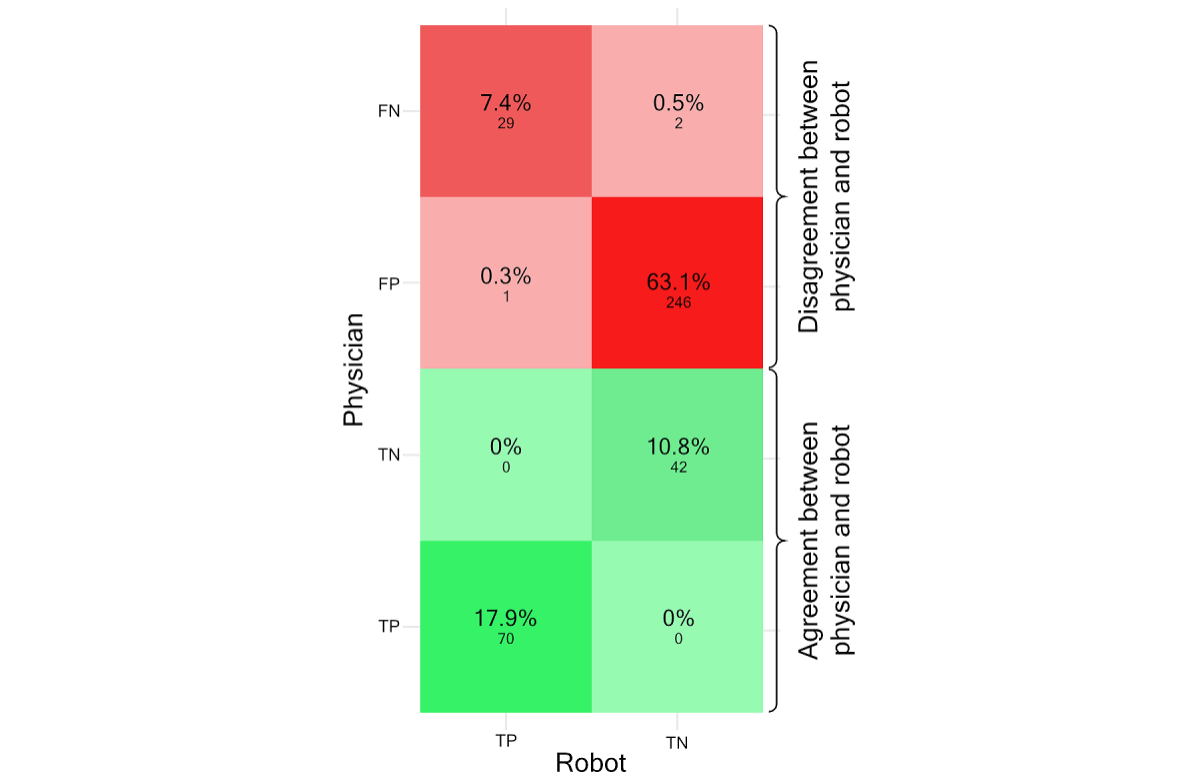
Confusion matrix of physician (predicted) and robot (actual) labels. Robot labels are judged as ground truth. Percentages of labels are shown in each square and the numbers of paired observations are below. FN: false negative; FP: false positive; TN: true negative; TP: true positive.

The primary finding of this study (Multimedia Appendix 2) was a significant difference in the ordered time interval to the next follow-up between robot 2 and the physician (robot: mean 4.5, SD 2.4 months; physician: mean 3.1, SD 1.4 months; *P<*.001). To analyze the model’s accuracy in predicting each class, paired orders from robot 2 and physicians (n=390 observations) were compared using a confusion matrix, which displays the actual versus predicted classifications. Based on robot 2 output defined as ground truth, the robot labels represent the actual values and the physician’s labels the predicted values (Figure 4). Of the observations, 112 (28.7%) corresponded between robot and physician. The most pronounced difference between the robot and the physician occurred for actual TNs according to the robot, which was judged as positive (predicted FP) by the physician in 246 (63.1%) observations.

**Figure 5:**
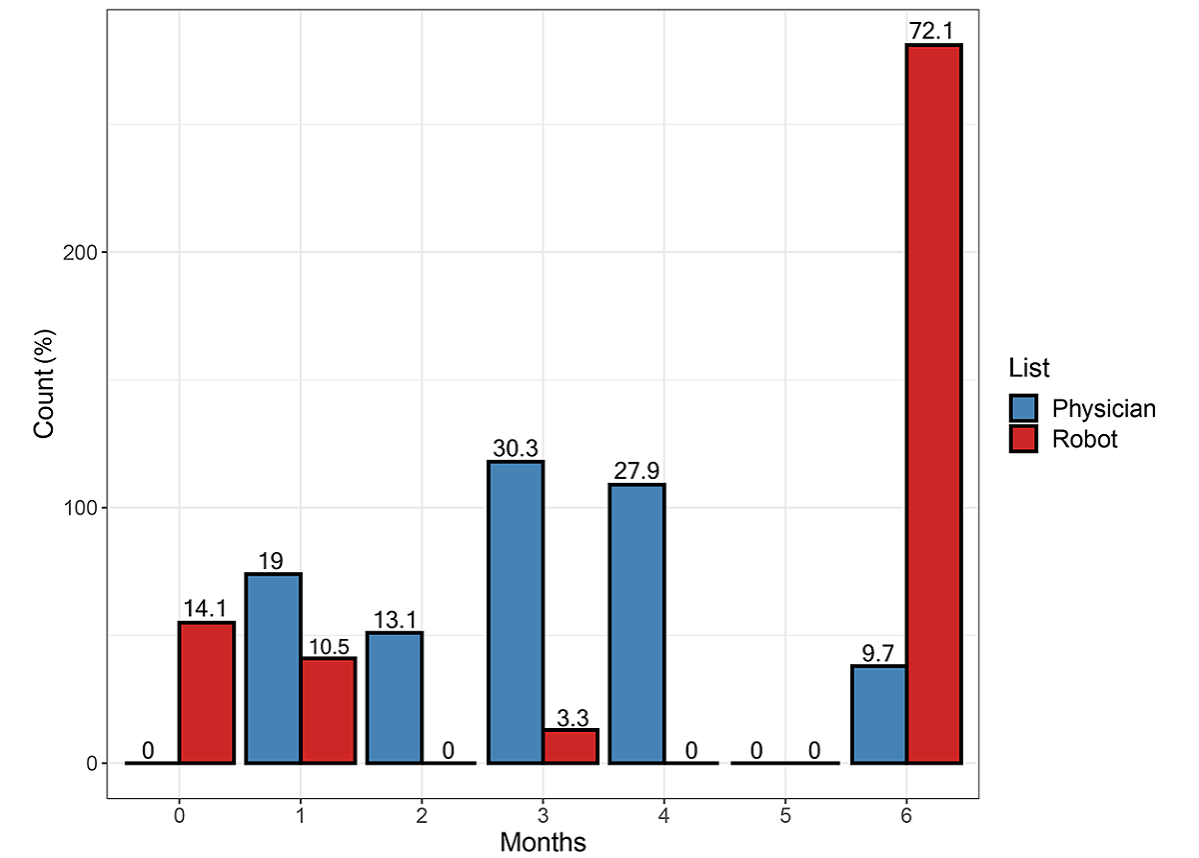
Bar chart of frequencies of patients who were ordered a time interval for follow-up by physician or robot, interval ranging from 0 to 6 months, on the x-axis. The absolute counts are shown on the y-axis and the percentage of physician and robot counts, respectively, is marked above the stacks (based on n=390 observations). For the robot, a red outcome implied a referral directly to the physician, which translated to “zero months.”.

The bar chart, illustrating the difference between robot 2 and the physician’s orders, demonstrates the largest visual difference in ordered times for follow-up as the robot ordered 6 months to the next control (ie, corresponding with normal laboratory values) in 281 (72.1%) observations, compared to physician’s orders in 38 (9.7%). The remaining robot orders were concentrated in lower time intervals (0 months, ie, recommending immediate physician’s action, and 1 month), whereas physician’s orders peaked at the 3-month interval (n=118, 30.3%), followed by 4 months (n=109, 27.9%; Figure 5).

**Figure 6:**
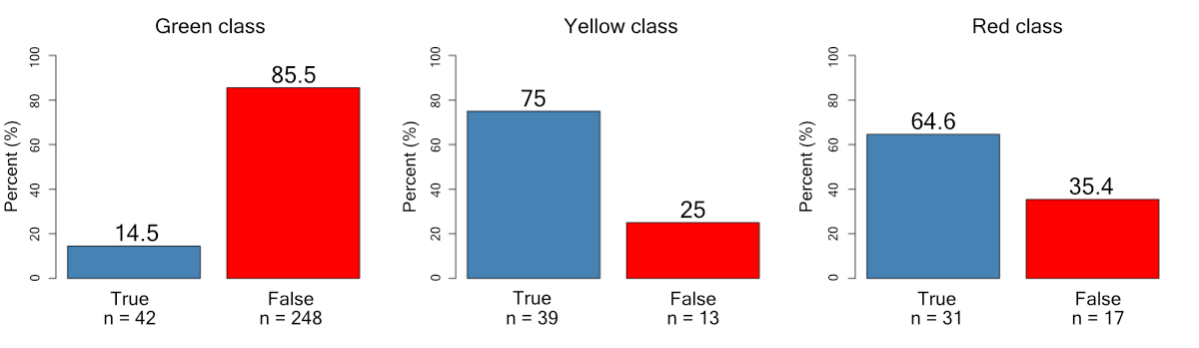
Discrepancies in outcome labels by the physician compared to the robot for the 3-color coded classes (representing different levels of aberration vs normality): (1) green class: normal laboratory values, (2) yellow class: minor deviation from normal or uncertain significance, (3) red class: definite laboratory pathology. “True” represents that the physician’s classification matched the robot (ground truth). “False” represents that the physician’s classification differed from ground truth. The height of the stacks represents the percentage of outcome labels (y-axis), and the absolute number of observations appears below the stacks.

Complementary analysis showed the largest discrepancy for cases with the lowest degree of urgency (class 1: green, follow-up in 6 months) where false decisions greatly outweighed the true ones. When more urgent action was called for (classes 2 and 3: yellow and red colors), the robot and physician orders were more aligned, especially for class red (clearly pathological findings; Figure 6).

### Detection of Side Effects

**Figure 7:**
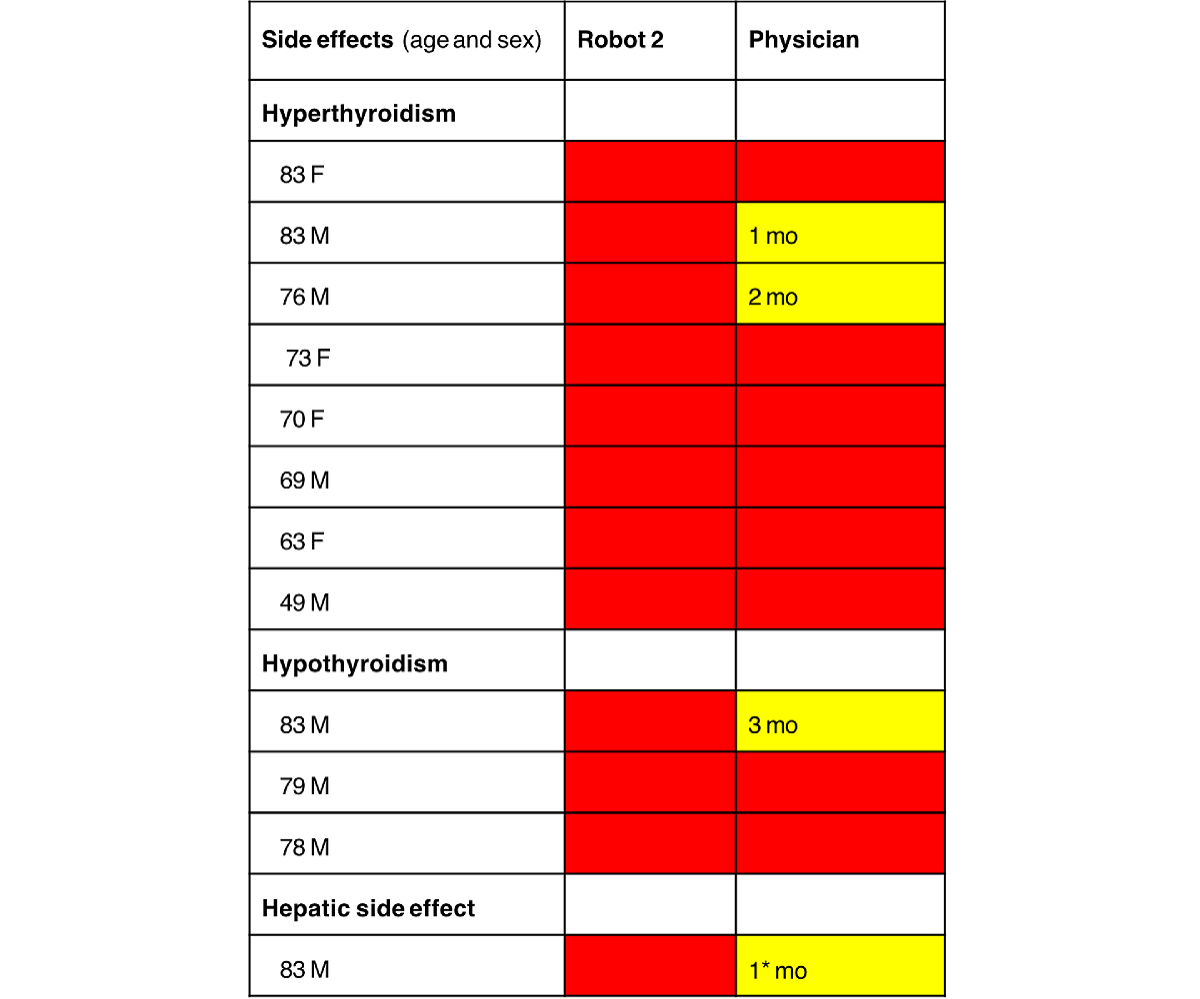
Diagnostic side effects during study of robot 2 and assessment by robot versus physician. Observation of a correct response to a side effect by the physician required that (1) the diagnostic side effect was noted in medical records by the physician, (2) the indication for amiodarone was reevaluated, (3) adjustment or termination of medication was undertaken, or (4) referral or consultation with an appropriate medical specialist was done. Incorrect or questionable action represented that new laboratory tests were ordered without any of these actions. For repeated laboratory tests only, the interval of months ordered is noted in the boxes. Observation of a correct response to a side effect (red) by the robot required that the outcome reading was of the highest alert (red) ordering referral to a physician for action. *The physician reduced the dosage of amiodarone and ordered repeated testing after 1 month but documented no rationale for the action. F: female; M: male; mo: months.

During the 1-year evaluation, 12 patients developed side effects detectable in laboratory analyses (AIT n=8, AIH n=3, and hepatic side effects n=1). The robot assessed all these as class 3 (red), that is, a correct diagnosis and action. The physician’s assessment was red in 8 and yellow in 4 (repeat ordering of follow-up laboratory tests after 1-3 months) of the corresponding cases (Figure 7).

## Discussion

### Overview

This study demonstrates that an automated process for decision-making based on thyroid and liver laboratory values outperformed a physician-operated manual routine for amiodarone follow-up, leading to reduced frequency of laboratory testing and more rapid detection of side effects. Amiodarone is the most effective antiarrhythmic drug to prevent recurrences of atrial fibrillation (AF) when other antiarrhythmic drugs have failed, are contraindicated, or when ablation of AF or ventricular arrhythmias is not an alternative [14]. In our cohort, AF constituted the most common arrhythmia. The efficiency of amiodarone paired with the considerable risk of side effects supports the rigorous follow-up routines prescribed in international recommendations [1,10], resulting in repetitive and time-consuming manual work in general cardiology practice.

Using automation through a software robot, an RPA, appears as one feasible solution to replace human labor and ensure compliance with guidelines. Software robots work with information systems via the front end, that is, via the presentation layer, and do not impact the underlying data infrastructure [15]. An RPA is suitable for a process that follows a standardized and rule-based structure (does not require cognitive or judgment effort), is conducted both often and manually by humans and requires multiple-system access [16]. Notably, this is not an artificial intelligence tool. The prototype provides recommendations to the physician as a human-in-the-loop. This retains the responsibility as a control mechanism, as all recommendations must be confirmed by physician’s orders. Human exception management is necessary to solve problems where cognition, intuition, and situational decisions are needed [17].

### Main Findings

Most notably, the robot ordered significantly longer intervals between tests compared to the physician which is especially pronounced in the low-risk cases. Using the robot may thus serve to reduce manual labor, lessen inconvenience and worry in patients, and liberate testing personnel for other care tasks. Additionally, the robot identified all laboratory-diagnosed side effects, as opposed to the physician, who initially missed 1 of 3. Thus, the use of the robot appeared to increase the safety of the procedure, based on more prompt recognition of side effects. Together, these findings support that monitoring recommendations based on appropriate expert clinical advice are superior to handling only by a cardiologist.

### Detailed Analysis

Based on the outcomes defined by ground truth, we found that the most prevalent disagreement between the robot and physician occurred when laboratory values were normal (TN), whereas the physician classified normal findings as pathological (FP). This resulted in physicians ordering a significantly shorter time for the next laboratory follow-up than as motivated by guidelines or laboratory findings. Interestingly, the physicians’ orders tended to cluster around the mean eligible choice, 3-4 months in almost 60%. This supposedly caused considerably more frequent laboratory follow-ups for patients when guided by the physician, leading to increased testing costs, manual labor, inconvenience, pain, and possible worries for the patients without contributing to a better quality of care.

In contrast, the robot-physician orders were better aligned for the more urgent laboratory results, indicating that clearly pathological findings would not be missed. Still, the detailed analysis showed that the robot was superior as it identified all side effects, compared to 1 of 3 missed at first presentation by the physician. Although the results appear indicative of a more rapid detection of side effects by the robot compared to the physician, this study was not designed or powered to evaluate the performance regarding the detection of side effects. As for the patterns of physician’s ordering, we can only speculate but lack empirical support. The shorter control intervals ordered, despite knowledge of guidelines, may reflect a precautionary principle and hesitation to choose intervals at the recommended scale’s margins. Other explanations may be decision fatigue and working under time constraints, which may result in excessive caution. The seemingly inferior detection of side effects by the physician may be due to the algorithm being based on expert clinical advice in thyroid and liver disease, and the physicians responsible for monitoring were cardiologists, not mastering the best practices in these disciplines.

The categorization of laboratory values based on an expert-guided algorithm, as created for robot 2 for validation, appeared crucial. Notably, the algorithm was specifically formulated by experienced clinicians with considerable experience in the laboratory effects of amiodarone medication, as no composite guidelines exist for this specific situation. This procedure secured appropriate recommendations for the management of pathological findings and also incorporated a margin for expected deviations due to amiodarone medication per se.

### Plans

Following the satisfying outcome, our process for monitoring amiodarone treatment has gained approval as an in-house manufactured medical device for introduction at our health care institution, the Sahlgrenska University Hospital, per Regulation 2017/745 of the European Parliament, Article 5(5) concerning medical devices [18]. We are currently implementing the robot in clinical practice, under follow-up and supervision. However, evolving technology presents new avenues for development, such as the integration of a locally hosted large language model in the robot for increased accuracy and clinical relevance. This would enable both compilation of laboratory outcomes and individual recommendations for the patient at hand, possibly also incorporating consideration of clinical information. Another goal, outside the scope of this study, is to perform a more detailed analysis of physicians’ behavior. This could be done through surveys or interviews with physicians, to explore the underlying reasons for not strictly following guidelines, such as stress or decision fatigue involved in decision-making in clinical situations.

### Strengths and Limitations

One strength of our study was that the robot was carefully iteratively tested and designed to address technical and medical issues before launching the optimized prototype, robot 2, for validation during a year of final testing. Thus, our study presents detailed information on feasibility and safety in a real-world setting. Notably, the procedure will only identify the most common side effects, detectable in blood laboratory tests, whereas the remaining risks must still be considered in clinical visits. The robot does not replace the clinical consultation but constitutes an automated safety net as clinical consultations are regularly less frequent than the need for follow-up laboratory testing.

One weakness was that the final testing was carried out in parallel with real-world monitoring, providing a simulated outcome as a randomized controlled trial was outside the scope of this study. Additionally, the sample size of 390 paired orders was relatively small, which may limit the generalizability and statistical power of the findings, although including a complete cohort of all patients receiving amiodarone prescriptions at our health care institution. For example, this study was not powered to evaluate the performance between methods regarding the detection of side effects, although the robot appeared to outperform physician recommendations. Additionally, the results imply positive health economic outcomes, which have not been explored in this study.

### Conclusion

This study aimed to design and test an automated process for amiodarone treatment monitoring, mastering the manual tasks, currently performed by nurses and physicians, with preserved patient value. After iterative design and technical improvements, a prototype for validation was tested for 1 year. We found that the automated process provided a reliable alternative to the present manual management of amiodarone treatment monitoring. The results indicated that the robot may reduce manual labor and frequency of laboratory testing and detect side effects with increased precision compared to the physician, thereby reducing costs, and enhancing patient value.

## Data Availability

The data underlying this paper cannot be publicly shared to protect the privacy of this study’s participants but will be made available upon reasonable request to the corresponding author. The software code for the RPA is available upon request to JL.

## Appendix

Multimedia Appendix 1 Table S1. Algorithm for thyroid and liver test results for the robotic process automation in treatment with amiodarone.

Multimedia Appendix 2 Brief PowerPoint (Microsoft Corp) description of study including illustration from interactive user interface.

## Abbreviations

AF: atrial fibrillation
AIH: amiodarone-induced hypothyroidism
AIT: amiodarone-induced thyrotoxicosis
FN: false negative
FP: false positive
RPA: robotic process automation
TN: true negative
TP: true positive
TSH: thyroid-stimulating hormone
