# Supplementary material for: Automated Process for Monitoring of Amiodarone Treatment: Development and Evaluation
Source: J Med Internet Res. 2025 Feb 19;27:e65473. doi: 10.2196/65473 (PMC11888117; doi:10.2196/65473)
Supplement: Multimedia Appendix 1 [file jmir_v27i1e65473_app1.docx]

**Supplemental information.**

**Development of an automated process for monitoring of amiodarone treatment** Birgitta I Johansson, Jonas Landahl, Karin Tammelin, Erik Aerts, Christina Lundberg, Martin Adiels, Martin Lindgren, Annika Rosengren, Nikolaos Papachrysos, Helena Filipsson Nyström, Helen Sjöland

**Table SI. Algorithm for thyroid and liver test results for the Robotic Process Automation in treatment with amiodarone**

**Clinical laboratory testing and reference values**

**Routine tests:** free thyroxine (fT4, reference range 9.8-17 pmol/L), thyroid stimulating hormone (TSH, reference range 0.4-3.7 mIU/L), aspartate aminotransferase (AST, reference range women 0.25-0.60 µkat/L, and men 0.25-0.75 µkat/L), and alanine transaminase (ALT, reference range women 0.15-0.75 µkat/L, and men 0.15-1.1 µkat/L).

**Additive tests:** free triiodothyronine (fT3 reference range 2.4-6.0 pmol/L), TSH-receptor antibodies (TRAb, reference range<1.8 IU/L), Thyreoperoxidase antibodies (TPO-ab, reference range <5.6 kIU/L)

**Algorithm for use by Robot 2** (units as disclosed above)

**Green:** Control in six months (in accordance with guidelines)

TSH 0.4 – 5.0

AST 0.25 – 0.75

ALT 0.15 – 1.10

**Commentary:** Here, a TSH level slightly above the reference range in the green group is justified for several reasons. Hypothyroidism develops gradually, and a value slightly outside the reference range is often transient, normalizing in 40-60% of cases (1). Current guidelines refer to a gray zone where treatment decisions remain uncertain (2-4). Also, this slight deviation does not worsen the arrythmia situation. Notably, this group also includes normal deviations caused by amiodarone treatment, such as increased fT4 with normal TSH levels due to impaired peripheral deiodination of fT4 to fT3, and occasionally slightly elevated TSH due to the Wolff-Chaikoff effect (5). Aminotransferase levels fall within reference ranges.

**Yellow:** Control of routine tests in one month

For TSH 0.1 –<0.4 or >5.0—9.0:

If TSH >5.0—9.0 and fT4 within range (9.8—17.0) Routine tests in one month + TPO-ab.

If TSH >5.0—9.0 and fT4 slightly decreased (8.0–<9.8) Routine tests in one month + TPO-ab.

If TSH 0,1—<0,4 and fT4 normal (9,8−17,0) Routine tests in one month + fT3 and TRAb. If fT3 elevated (>6,0) or TRAb elevated (≥1.8) go to Red.

If “control in one month” is ordered at three times, escalate to Red.

If AST >0.75−≤2.0: Control of AST and ALT in one month, if AST >0.75−≤2 control of AST and ALT in three months. If remaining at the same level, continue every third months.

If ALT>1.1−≤2.0: Control of AST and ALT in one month, if ALT >1.1−≤2 control of AST and ALT in three months. If remaining at the same level, continue every third months.

**Commentary:** The yellow group includes test outcomes deviating slightly more from reference values than those in the green group, but still allowing for sufficient time to recheck. Following the same reasoning as in the green group, TSH 5.0-9.0 mIU/L will not necessarily require immediate treatment. Also, the diagnosis of subclinical or overt hypothyroidism due to thyroid disease should be strengthened by the presence of TPO antibodies. However, the levels motivate a heightened awareness to capture possible worsening, motivating follow-up at a shorter time interval than 6 months, to comply with clinical practice.

This group also includes the indecisive zone of TSH 0.1-0.4 mIU/L, which, according to present guidelines for subclinical hyperthyroidism, requires rechecking along with fT3 and TRAb testing (2-4) to detect overt hyperthyroidism and/or Graves’ disease. The proposed procedure will not delay accurate management.

For amiodarone, liver serum enzyme elevations are reported in 15% to 80% of patients on long-term therapy. They often resolve spontaneously even if amiodarone is continued. Liver biopsy findings in this group has minimal changes and typically without significant tissue damage. However, persistent elevations warrant closer monitoring (6-8).

**Red:** Directly for assessment by physician

TSH <0,4 and elevated fT4 (>17.0)

TSH <0.1 and fT4 within range (9.8─17.0)

TSH >5.0─9.0 and low fT4 (<8.0)

TSH >9.0

AST >2.0

ALT >2.0

**Commentary:** For the cases above, overt hyperthyroidism or definite subclinical hyperthyroidism (with TSH <0.1 mIU/L) (rows 1-2), the subject will be managed directly by the physician with a recommendation of referral to an endocrinologist, especially as this situation may increase the arrythmias. However, most often the decreased peripheral conversion of fT4 to fT3 during amiodarone treatment limits that risk (5). Similarly in cases with overt hypothyroidism or with high TSH (rows 3-4) we would not want to delay management within primary care. Clinically apparent liver disease occurs in up to 1% of patients treated with amiodarone annually. The risk increases with higher doses or prolonged treatment. A significant elevation in aminotransferases (rows 5-6) requires immediate physician assessment to exclude clinically apparent liver disease and determine whether amiodarone therapy should be discontinued (9-10).

**Instruction to cardiologist handling test results classified as “Red”**

Assessment by physician always implies that the cardiologist evaluates the indication for amiodarone, and possibly adjusts the dose.

For specific results:

| **Hyperthyroidism** (results as below):  TSH <0.4 and elevated fT4 (>17.0).  TSH <0.1 and fT4 within range.  If TSH 0.1–0.4 and fT4 within range (9.8−17.0) (subclinical hyperthyroidism) and elevated fT3 (>6.0) or elevated TRAb (≥1.8).  **Hypothyroidism** (results as below):  TSH >5.0−9.0 and decreased fT4 (<8.0)  TSH >9.0  If TSH >5.0−9.0 and fT4 within range  (9.8−17.0) and elevated TPO-ab (≥5.6).  If TSH >5.0−9.0 and fT4 slightly decreased (8.0−<9.8) and elevated TPO-ab (≥5.6).  **Liver injury**  Persistent AST and ALT > 2.0, up to 5x upper normal limit.  AST and ALT >5 times above upper limit.  AST and ALT >10 times above upper limit | Discontinue medication and immediately notify endocrinologist.  Order TSH, fT4, fT3 and TRAb within 2 weeks, simultaneously notify endocrinologist. Hold off change of medication.  Referral to endocrinologist. Consider change in medication.  Consider levothyroxine and indication for amiodarone. Refer to endocrinologist.  As above.  Subclinical hypothyroidism. TPO-ab explains the mechanism. If no symptoms, continue every third months. If symptomatic refer to  primary care.  Consider low dose levothyroxine. Consider indication for amiodarone treatment. If symptomatic refer to primary care.  Consider discontinuation or pausing of treatment. Reinstatement of low dose may be considered after normalization of AST and ALT.  Referral to hepatologist.  Immediately contact hepatologist. |
| --- | --- |

**References:**

1. van der Spoel E, van Vliet NA, Poortvliet RKE, Du Puy RS, den Elzen WPJ, Quinn TJ, Stott DJ, Sattar N, Kearney PM, Blum MR, Alwan H, Rodondi N, Collet TH, Westendorp RGJ, Ballieux BE, Jukema JW, Dekkers OM, Gussekloo J, Mooijaart SP, van Heemst D. Incidence and Determinants of Spontaneous Normalization of Subclinical Hypothyroidism in Older Adults. J Clin Endocrinol Metab. 2024 Feb 20;109(3):e1167-e1174. doi: 10.1210/clinem/dgad623. PMID: 37862463; PMCID: PMC10876405.
2. Kahaly GJ, Bartalena L, Hegedus L, Leenhardt L, Poppe K, Pearce SH. 2018 European Thyroid Association Guideline for the Management of Graves' Hyperthyroidism. European thyroid journal 2018; 7: 167-86.
3. Ross DS, Burch HB, Cooper DS, et al. 2016 American Thyroid Association Guidelines for Diagnosis and Management of Hyperthyroidism and Other Causes of Thyrotoxicosis. Thyroid 2016; 26: 1343-421.
4. https://vardpersonal.1177.se/globalassets/nkk/nationell/media/dokument/kunskapsstod/vardprogram/nationellt-vardprogram-for-hypertyreos.pdf
5. Bartalena L, Bogazzi F, Chiovato L, Hubalewska-Dydejczyk A, Links TP, Vanderpump M. 2018 European Thyroid Association (ETA) Guidelines for the Management of Amiodarone-Associated Thyroid Dysfunction. European thyroid journal 2018; 7: 55-66.
6. Zimmerman HJ. Amiodarone. Drugs used in cardiovascular disease. In, Zimmerman HJ. Hepatotoxicity: the adverse effects of drugs and other chemicals on the liver. 2nd ed. Philadelphia: Lippincott, 1999, pp. 648-52.
7. Kowey PR, Friehling TD, Marinchak RA, Sulpizi AM, Stohler JL. Safety and efficacy of amiodarone. The low-dose perspective. Chest 1988; 93: 54-9.
8. Lewis JH, Ranard RC, Caruso A, Jackson LK, Mullick F, Ishak KG, Seeff LB, Zimmerman HJ. Amiodarone hepatotoxicity: prevalence and clinicopathologic correlations among 104 patients. Hepatology 1989; 9: 679-85.
9. De Marzio DH, Navarro VJ. Amiodarone. Hepatotoxicity of cardiovascular and antidiabetic drugs. In, Kaplowitz N, DeLeve LD, eds. Drug-induced liver disease. 3rd ed. Amsterdam: Elsevier, 2013, pp. 520-1.
10. Fogoros RN, Anderson KP, Winkle RA, Swerdlow CD, Mason JW. Amiodarone: clinical efficacy and toxicity in 96 patients with recurrent, drug-refractory arrhythmias. Circulation 1983; 68: 88-94.
