# Supplementary material for: Automated Process for Monitoring of Amiodarone Treatment: Development and Evaluation
Source: J Med Internet Res. 2025 Feb 19;27:e65473. doi: 10.2196/65473 (PMC11888117; doi:10.2196/65473)
Supplement: Multimedia Appendix 2 [file jmir_v27i1e65473_app2.pdf]

An automated process for monitoring of amiodarone treatment: Development and evaluation

Helen Sjöland, MD, PhD

UNIVERSITY OF GOTHENBURG

VÄSTRA GÖTALANDSREGIONEN
SAHLGRENSKA UNIVERSITETSSJUKHUSET

Follow-up of side-effects of medication

- Amiodarone (Cordarone) very effective antiarrhythmic substance
- Long-term treatment associated with high risk of side-effects (thyroid and liver)
- Need for repeated laboratory controls ”Cordarone clinics”
- Completely manual handling

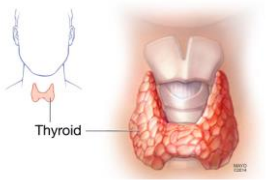

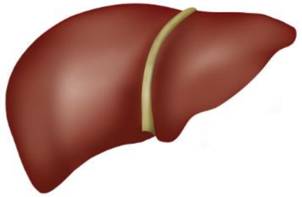
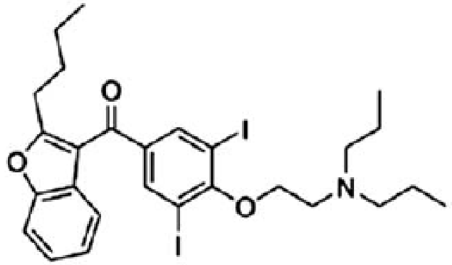

From idea to implementation

- Process mapping
- Software robot model (RPA)
- **First prototype** 2021 (Robot 1): Test run with iterative adjustments
- **Final prototype in ”silent trial”** 2022 (Robot 2): Test run in parallel with present manual routine
- Analysis and evaluation
- Implementation

Decision algorithm ”Human in the loop”

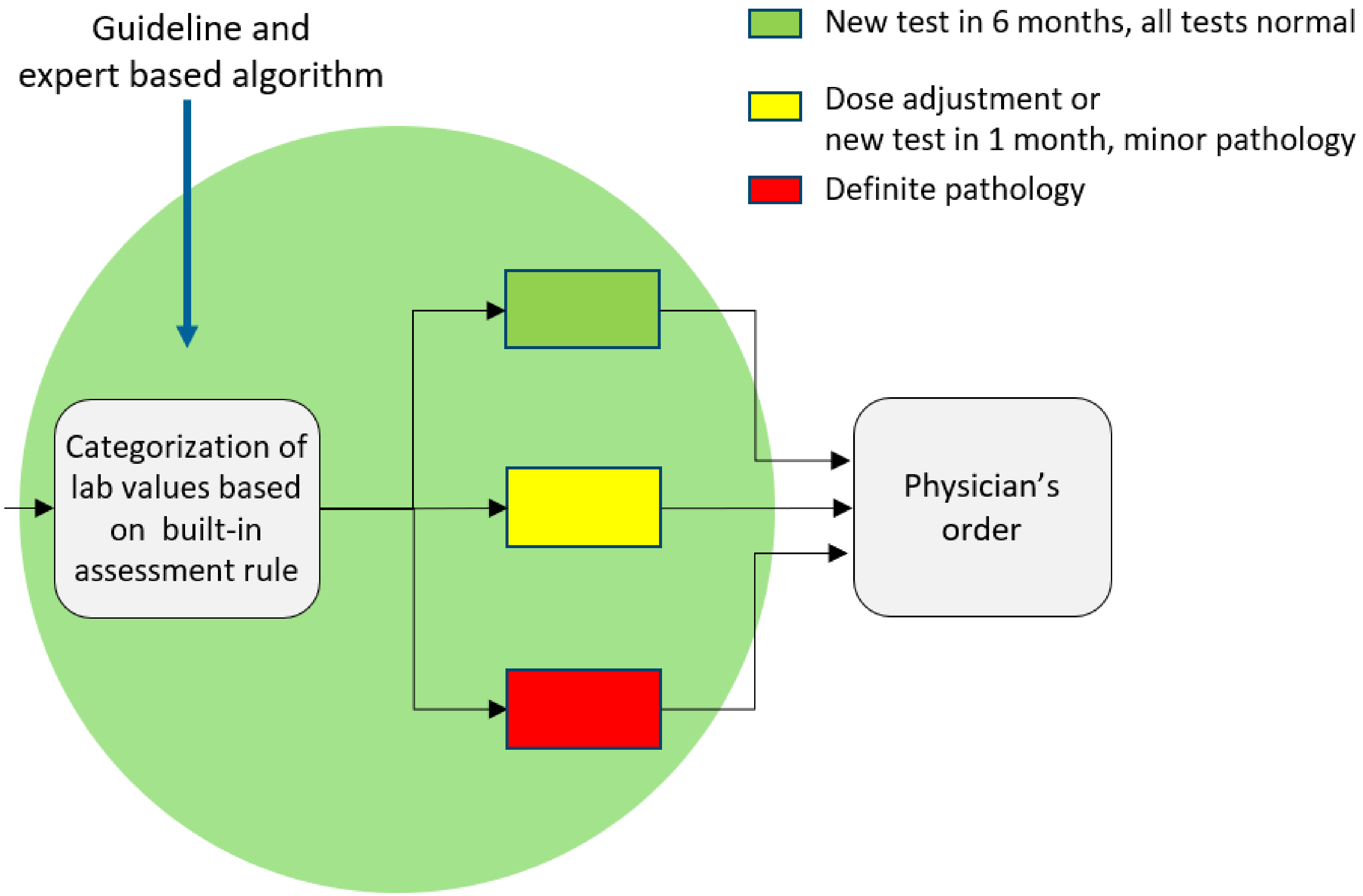

Actions

- [green] Normal outcome, new test 6 months
- [yellow] Small/uncertain deviation, new test 1 months
- [red] Definite pathology, to physician for actions defined in user manual

Interactive user interface

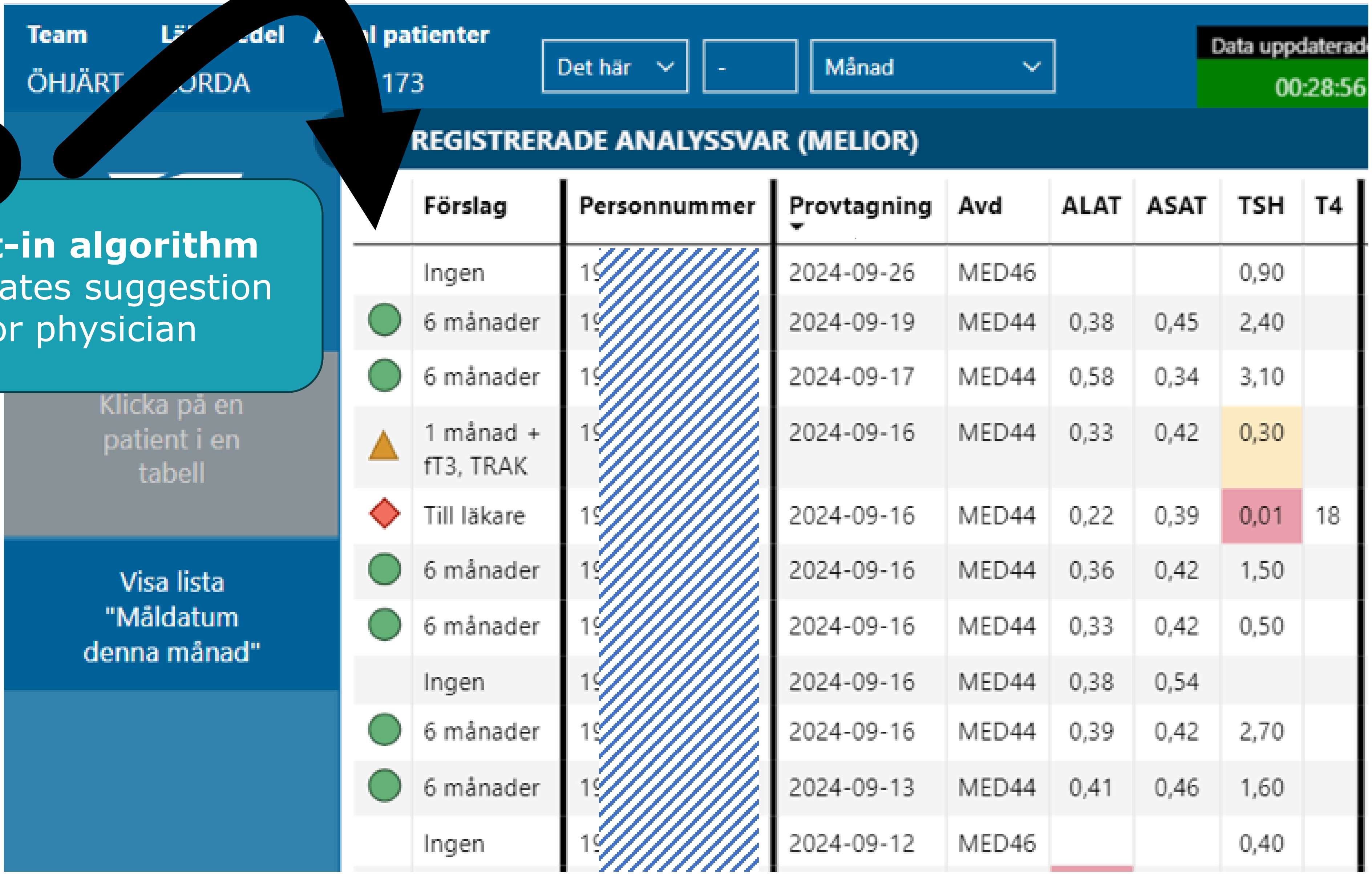

Förslag	Personnummer	Provtagning	Avd	ALAT	ASAT	TSH	T4
Ingen	19[illegible]	2024-09-26	MED46			0,90	
6 månader	19[illegible]	2024-09-19	MED44	0,38	0,45	2,40	
6 månader	19[illegible]	2024-09-17	MED44	0,58	0,34	3,10	
1 månad + fT3, TRAK	19[illegible]	2024-09-16	MED44	0,33	0,42	0,30	
Till läkare	19[illegible]	2024-09-16	MED44	0,22	0,39	0,01	18
6 månader	19[illegible]	2024-09-16	MED44	0,36	0,42	1,50	
6 månader	19[illegible]	2024-09-16	MED44	0,33	0,42	0,50	
Ingen	19[illegible]	2024-09-16	MED44	0,38	0,54		
6 månader	19[illegible]	2024-09-16	MED44	0,39	0,42	2,70	
6 månader	19[illegible]	2024-09-13	MED44	0,41	0,46	1,60	
Ingen	19[illegible]	2024-09-12	MED46			0,40	

1

Built-in algorithm generates suggestion for physician

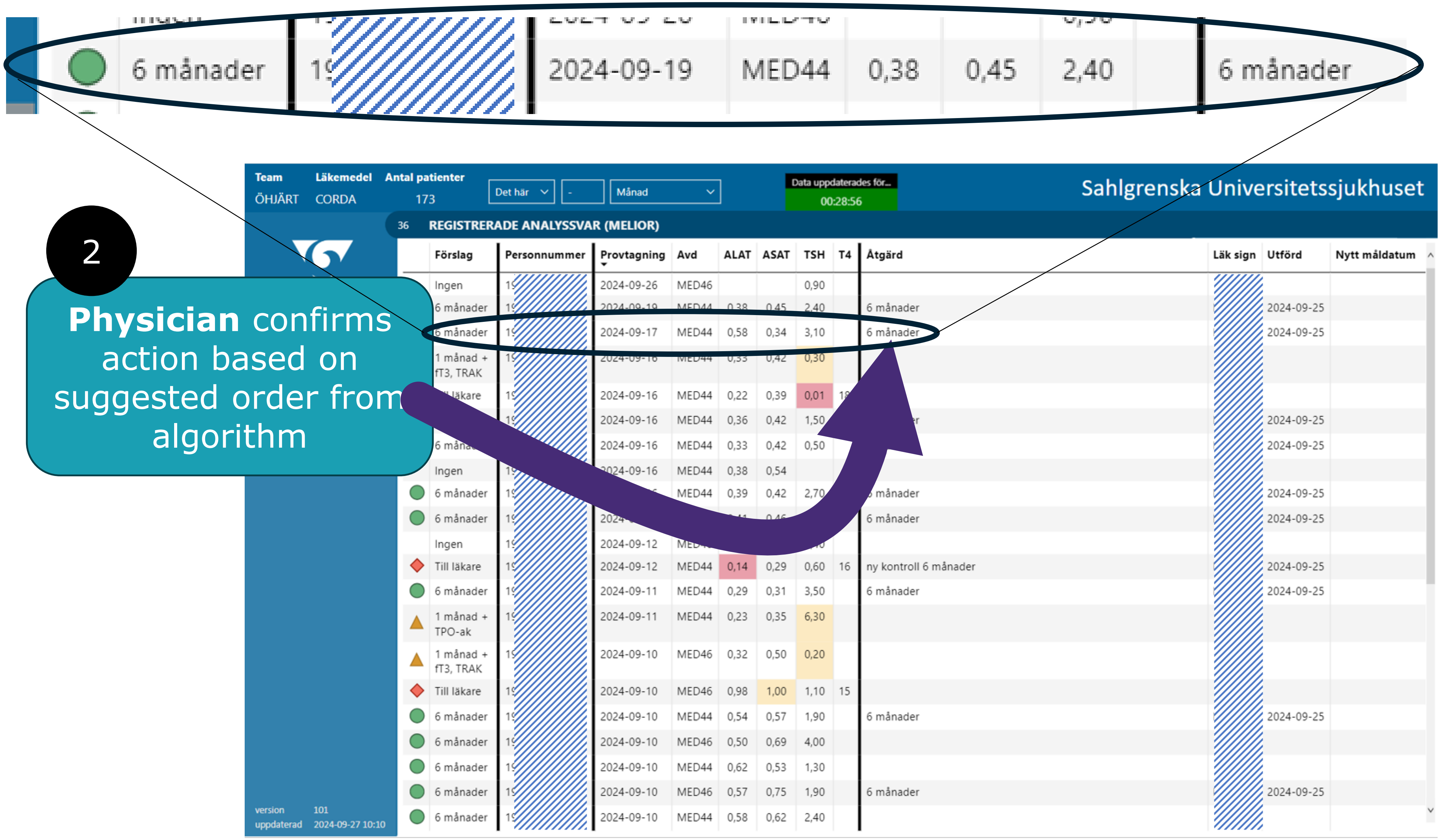

2

Physician confirms action based on suggested order from algorithm

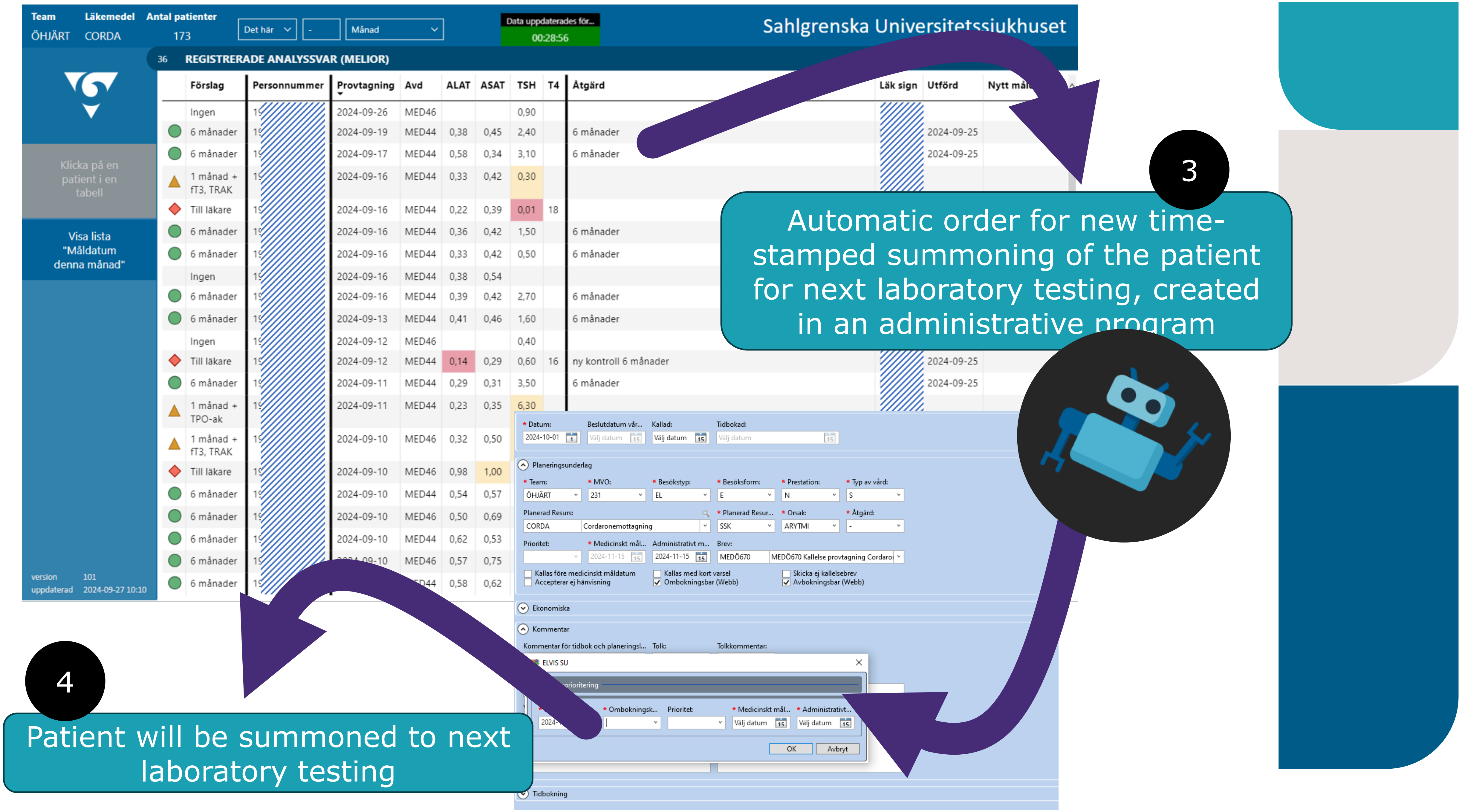
Team
ÖHJÄRT
Läkemedel
CORDA
Antal patienter
173
Det här
Månad
Data uppdaterades för...
00:28:56
Sahlgrenska Universitetssjukhuset
36 REGISTRERADE ANALYSSVAR (MELIOR)
Klicka på en patient i en tabell
Visa lista "Måldatum denna månad"
3
Automatic order for new time-stamped summoning of the patient for next laboratory testing, created in an administrative program
4
Patient will be summoned to next laboratory testing
